# The Photothermal Effect of Targeted Methotrexate-Functionalized Multi-Walled Carbon Nanotubes on MCF7 Cells

**DOI:** 10.22037/ijpr.2020.14484.12423

**Published:** 2019

**Authors:** Ali Karimi, Mohammad Erfan, Seyed Alireza Mortazavi, Fatemeh Ghorbani-Bidkorbeh, Behnaz Landi, Farzad Kobarfard, Farshad H. Shirazi

**Affiliations:** a *Department of Pharmaceutics, School of Pharmacy, Shahid Beheshti University of Medical Sciences, Tehran, Iran. *; b *Department of Medicinal Chemistry, School of Pharmacy, Shahid Beheshti University of Medical Sciences, Tehran, Iran.*; c *Department of Toxicology and Pharmacology, School of Pharmacy, Shahid Beheshti University of Medical Sciences, Tehran, Iran.*

**Keywords:** Multi-walled carbon nanotubes, Photothermal, Folic acid, Methotrexate, Polyethylenimine, Targeting effect; Cancer cell

## Abstract

Our goal is to reduce the release rate of methotrexate (MTX) and increase cell death efficiency.Carboxylated multi-walled carbon nanotubes (MWCNT-COOH) were functionalized with MTX as a cytotoxic agent, FA as a targeting moiety and polyethylene amine (PEI) as a hydrophilic agent. Ultimately, MWCNT-MTX and MWCNT-MTX-PEI-FA were synthesized. Methotrexate release studies were conducted in PBS and cytotoxic studies were carried out by means of the MTT tassay.

Methotrexate release studies from these two carriers demonstrated that the attachment of PEI-FA onto MWCNT-MTX reduces the release rate of methotrexate. The IC50 of MWCNT-MTX-PEI-FA and MWCNT-MTX have been calculated as follows: 9.89 ± 0.38 and 16.98 ± 1.07 µg/mL, respectively. Cytotoxic studies on MWCNT-MTX-PEI-FA and MWCNT-MTX in the presence of an IR laser showed that at high concentrations, they had similar toxicities due to the MWCNT’s photothermal effect. Targeting effect studies in the presence of the IR laser on the cancer cells have shown that MWCNT-MTX-PEI-FA, MWCNT-MTX, and f-MWCNT have triggered the death of cancer cells by 55.11 ± 1.97%, 49.64 ± 2.44%, and 37 ± 0.70%, respectively.

The release profile of MTX in MWCNT-MTX-PEI-FA showed that the presence of PEI acts as a barrier against release and reduces the MTX release rate. In the absence of a laser, MWCNT-MTX-PEI-FA exhibits the highest degree of cytotoxicity. In the presence of a laser, the cytotoxicity of MWCNT-MTX and MWCNT-MTX-PEI-FA has no significant difference. Targeting studies have shown that MWCNT-MTX-PEI-FA can be absorbed by cancer cells exclusively.

## Introduction

Methotrexate (MTX) is a folic acid antagonist that attaches to the dihydrofolate reductase enzyme and prevents the synthesis of purine and pyrimidine nucleotides and therefore prevents the production of DNA and RNA. At pH = 6, a very strong bond between methotrexate and the enzyme is established. Methotrexate has many side effects, including bone marrow suppression, alopecia and stomatitis (1, 2).

To reduce side effects and lessen damage to healthy tissues and cells, we are forced to reduce the MTX dose. This reduces the effectiveness of the treatment. The best method to reduce side effects and increase the therapeutic efficacy is targeted drug delivery to cancerous cells. There are two mechanisms for drug targeting: passive and active targeting. In passive drug targeting, depending on the size of the particles, the drug accumulates in the tumor. Cancerous tissues have tortuous and poorly differentiated vasculature compared with healthy tissues. Therefore, larger-sized drugs can penetrate the tumor vasculature but not the healthy tissue vasculature. Therefore drug side effects are reduced. This method is called enhanced permeability and retention (EPR). In active targeting, the selected ligands belong to receptors that are over-expressed on cancerous cells and so these ligands are absorbed by their receptors on the surface of the cancerous cells (3, 4). There are various ligands utilized for drug targeting to cancer cells. These ligands include: antibodies (5), peptides (6) and folic acid (7). The drug and the ligand are placed on a carrier and sent to the cancer cells. There are many carriers for this purpose, such as polymers, liposomes, dendrimers, carbon materials, and magnetic nanoparticles (8). Recently, carbon nanotubes have attracted much attention in the field of tissue engineering (9-11), medicine, and pharmaceutical sciences (12-14). The reason for this considerable interest towards carbon nanotubes is its numerous positive properties. These properties include photoluminescence properties, photothermal effect, high surface area, high chemical stability, and not to mention the ease with which the carbon nanotube surface can be functionalized. One of the benefits of using carbon nanotubes is that its surface can be functionalized without much difficulty. There are two general methods for surface modification of carbon nanotubes: the non-covalent (15) and covalent methods. Non-covalent bonding onto the carbon nanotube surface is accomplished by three mechanisms: hydrophobic interactions, π-π interactions and electrostatic interactions. π -π interactions are between two compounds with aromatic rings (16). Wang and coworkers investigated the adsorption of methylene blue and acid dye (acid red 183, AR183) onto the surface of carbon nanotubes. Adsorption of these compounds onto the surface of carbon nanotubes is accomplished through the π-π interaction. In single dye systems, the maximum adsorption capacities of methylene blue and AR183 on the CNT surface was 59.7 and 45.2 mg/g, respectively (17). Yang and coworkers studied the π-π interactions between the carbon nanotubes and aromatic amino acids such as phenylalanine, tyrosine, and tryptophan by quantum mechanical and molecular mechanical calculations. These calculations reveal *π*-*π *interactions between the CNT and amino acids (18). Hydrophobic interactions occur between the hydrocarbon chains of a compound and the hydrophobic surface of the carbon nanotube (19-21). MWCNT-COOH surface has a negative charge and can electrostatically conjugate to the cationic polymers and compounds. These types of interactions are called electrostatic interactions (22, 23). Nengqin and coworkers attached paclitaxel (PTX) to the carbon nanotube surface through π-π interactions, and then the cationic PEI polymer was affixed to the MWCNT-PTX surface through electrostatic interactions and finally, folic acid was coupled to PEI via covalent bonding. This carrier displayed significant cytotoxic effect on the Hela cells compared with PTX alone (16). In the covalent technique, various types of compounds are covalently bonded to the carbon nanotube surface (24). Antonio and coworkers conjugated carboxylated MWCNT to polyethylenimine (PEI), polyallylamine (PAA), or a mixture of the two polymers, covalently, with the intention of transferring plasmid DNA *in-vitro* (25). Sanyog Jain and coworkers connected 2,2-(ethylene dioxy)bis-(ethylene amine) (EDBE) to carboxylated carbon nanotubes by using tionyl chloride then folic acid and methotrexate through N-hydroxysuccinimide (NHS) ester and carbodiimide chemistry connected to EDBE (7). Another important property of carbon nanotubes is their photoluminescence properties. After laser irradiation, carbon nanotubes have a very large emission range from 1100 to 1400 nm, which can be detected by the detector in different tissues. This feature is very important in *in-vivo* studies and in-vivo imaging (21).

Another important feature of carbon nanotubes is their photothermal effect. Carbon nanotubes are able to absorb a wide range of light from ultraviolet to infrared (IR) and convert it to heat. Biological systems are not able to absorb near IR (700 to 1,100 nm) (26). Therefore, we can send carbon nanotubes to the desired location and irradiate it with near IR radiation and raise the temperature of the location. This property can be used to kill cancer cells (27-30). The amount of heat generated by carbon nanotubes depends on the type of carbon nanotube, its concentration, the intensity of the laser, and the amount of time of exposure to radiation. Multi-walled carbon nanotubes produce more heat than single-walled carbon nanotubes (31). By increasing the concentration of the carbon nanotube, the strength of the laser and the radiation time, the amount of heat generated is increased (26, 31, 32).

In this paper, methotrexate and FA were attached to the MWCNT surface and PEI, respectively, by covalent bond and MWCNT-MTX and PEI-FA were produced. Then MWCNT-MTX was attached to PEI-FA by way of electrostatic interactions and MWCNT-MTX-PEI-FA was produced. The cytotoxic effects of MWCNT-MTX and MWCNT-MTX-PEI-FA were studied on the MCF7 cancer cells in the presence and absence of an IR laser. Subsequently, in the presence of the IR laser, the targeting effects of MWCNT-MTX-PEI-FA were studied on MCF7 cancer cells. Lastly, the release profiles of MWCNT-MTX and MWCNT-MTX-PEI-FA were analyzed using a spectrophotometric method.

## Experimental

*Materials*

MWCNTs (Purity:   > 90 wt% , outside diameter: 10-30 nm, inside diameter: 5-10 nm, length: 10-30 μm) were **purchased** from US research nanomaterials. N-hydroxysuccinimide (NHS), N,N’-Dicyclohexylcarbodiimide (DCC), metho-trexate (MTX), 1-Ethyl-3-(3-dim-ethylaminopropyl)carbodiimide (EDC), folic acid (FA)**, **polyethylenimine (PEI), branched (average M_w_ ~25,000, average M_n_ ~10,000, branched), Trypsin and 3-(4,5-dimethylthiazol-2-yl)-2,5-diphen-yltetrazolium bromide (MTT) were **purchased** from Sigma Aldrich. The dialysis bags (MWCO = 12,000) were purchased from Spectrum Laboratories Inc. 808 nm continuous wave NIR diode laser beam (1.5 W/cm^2^) were purchased from BWT Beijing LTD, Beijing, China. All cell lines were grown in RPMI medium and supplemented with 10% heat inactivated fetal bovine serum, antibiotics: penicillin, streptomycin (all chemicals from Sigma-Aldrich, St. Louis, MO, USA).

*Purification and Carboxylation of WCNTs*

500 mg of the MWCNTs were purified and carboxylated using a mixture of nitric acid and sulfuric acid (1:3 v/v) in 80 °C and mixed for 6 h . The mixture resulting from the reaction involving the carboxylation of MWCNTs (f-MWCNTs) was washed to neutrality with deionized water and then it was centrifuged with ultracentrifuge (BECKMAN COULTER, optima L-90K) for 30 min and 40000 rpm to separate the f-MWCNTs as powder. The MWCNTs and the f-MWCNTs were characterized using Raman spectroscopy (SENTERRA, BRUKER, Germany, Spectral range: 200-3500 cm^-1^, laser wavenumber: 785 nm), thermogravimetric analyzer (TGA, Shimadzu, Japan) and elemental analysis. The size and structures of the f-MWCNTs were observed using a FE-SEM microscope (Hitachi S-4160, Japan) (27). 

*Synthesis and purification of the MWCNT-MTX*

**Scheme 1:**
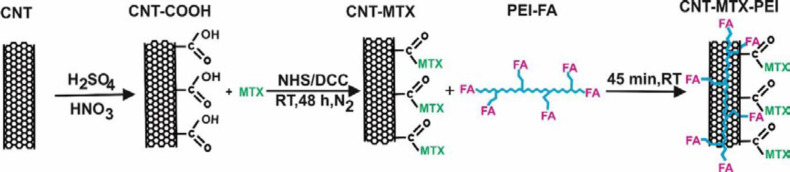
Functionalization of f- MWCNT with MTX, PEI and FA

1442 mg DCC, 805 mg NHS and 250 mg f-MWCNT reacted with each other in DMSO for 48 h in dark conditions, at room temperature and a nitrogen atmosphere and dicyclohexylurea precipitates and MWCNT-NHS were produced. The dicyclohexylurea was filtered and 125 mg MTX was added to the filtered mixture. MTX and MWCNT-NHS underwent a chemical reaction for 48h in dark conditions at room temperature and MWCNT-MTX was produced (Scheme 1). 90 mL of water was added to the solution to precipitate MWCNT-MTX, and solvents and other impurities were separated from the MWCNT-MTX by means of dialysis bags in water (7).

*Synthesis and purification of the MWCNT-MTX-PEI-FA*

140 mg EDC and 200 mg FA underwent a reaction in water for 1h in dark conditions at room temperature and FA-EDC was synthesized. Then 1000 mg PEI was added to the solvent for 48h in dark conditions at room temperature, and PEI-FA was produced. 140 mg CNT-MTX and 40 mg PEI-FA were mixed for one hour and CNT-MTX-PEI-FA was synthesized (Scheme 1) (7, 16).

*Release studies of MTX in MWCNT-MTX and MWCNT-MTX-PEI-FA*

4 mg each of MTX, MWCNT-MTX and MWCNT-MTX-PEI-FA were dispersed separately in 1 mL phosphate buffer apiece which were then transferred to three individual dialysis bags (cut-off = 12000) and dialyzed in 40 mL of phosphate buffer (0.1 M, H=7.4 and 37 ± 3 °C), for each. The dialysis solution was changed several times during the test to allow the sink conditions to be maintained. The dialysis solution was sampled at different time intervals, and using the MTX calibration curve at 300 nm, the concentration of released methotrexate was calculated using a UV-visible spectrophotometer (RAY LEIGH, UV-2601, China). Methotrexate has a maximum absorbance at 300 nm. The PBS was refreshed at specified time intervals. Release studies were repeated three times.

*Cytotoxicity studies in the presence and absence of a laser*

Breast cancer cell lines (MCF7) were cultured in T25 flasks in RPMI Media which were supplemented with 10% fetal bovine serum (FBS) and 1% antibiotics penicillin and streptomycin. Cells were passaged three times to reach normal mode before the experiment. On the day of the experiment 6000 cells per well were cultured in two **96** well **plates.** After 24 h, when cell adherence to the bottom of the flask was established, 20 micro-liters MTX, f-MWCNT, MWCNT-MTX and MWCNT-MTX-PEI-FA were added to each well in the final concentrations of 5, 10 and 20 micrograms per mL respectively. The concentrations were calculated based on MTX content of the nanotube. The first plate was incubated for 48 h at 37 °C and 5% CO_2_. Cells in the second plate were irradiated for 3 min with an 808 nm laser. Laser beam size was 0.5×0.7 cm and its intensity was 1.5 W/cm^2^. Second plate was then incubated for 48 h at 37 °C and 5% CO_2_. Subsequently the cell death for each well was analyzed using the MTT assay and Enzyme-Linked Immuno Sorbent Assay (ELISA, 808 IU, BioTek). Cytotoxicity studies were repeated six times (32).

*Targeting studies in the presence of a laser*

In order to evaluate the targeting effect, 6000 MCF7 cells were cultured per well. The cells were incubated for 24 h at 37 °C. Next, 20 microliters each of f-MWCNT, MWCNT-MTX and MWCNT-MTX-PEI-FA in final concentrations of 20 micrograms per milliliter was added to each well. Then MCF7 cells were incubated for 3 h. The cells were washed twice with normal saline to eliminate the carriers not attached to the cells. Thereafter each well was irradiated for 3 min with an 808 nm laser. Finally after 48 h, the cell death was analyzed using the MTT assay (28).

*Statistical studies*

To investigate the significance of cellular cytotoxicity in the presence and absence of laser, SPSS software (SPSS statistics 17.0) was used. For this purpose, one way ANOVA test with confidence intervals of 95% was used.

## Results and Discussion

*Characterization of MWCNT-MTX and MWCNT-MTX-PEI-FA*

**Figure 1:**
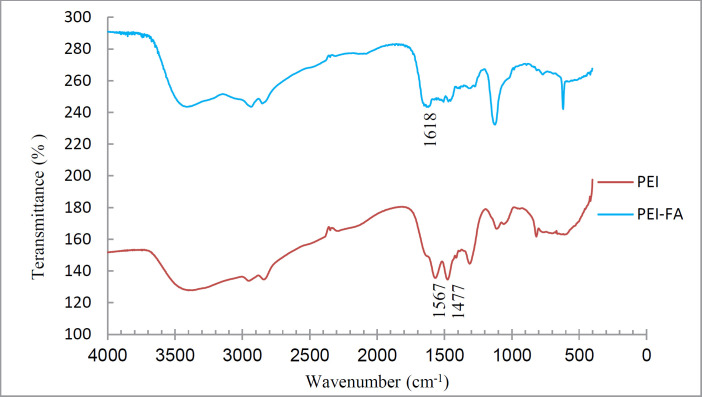
IR spectrum of PEI and PEI-FA

To ensure the coupling of FA to PEI, the IR spectra of PEI-FA and PEI were taken. Figure 1 shows the IR spectra of PEI and PEI-FA samples. The peak located at 1618 in the PEI-FA spectrum is attributable to an amide bond. Peaks at 1477 and 1567 in the PEI spectrum -which are eliminated in the PEI-FA spectrum- are due to N-H amine. This indicates that the PEI amines reacted with FA carboxyl and were converted to an amide bond.

**Figure 2:**
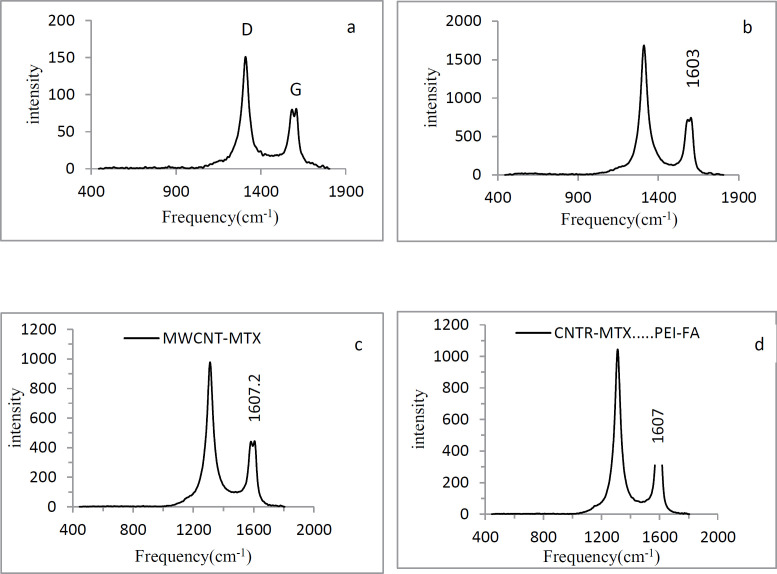
Raman spectra of naked MWCNT(a), f-MWCNT (b), MWCNT-MTX(c) and MWCNT-MTX-PEI-FA(d).

Carbon atoms in the MWCNT surface have SP^2^ hybridization. With the carboxylation of carbon nanotubes, the amount of SP^3^ carbon on the f-MWCNT surface increases. The Raman spectra of MWCNT has two index peaks: D and G peaks. The D peak is located at 1300 cm^-1^ and reflects the amount of SP^3^ carbon on the MWCNT surface. As the amount of SP^3^ carbon increases, the D peak intensity increases. The G peak is located at 1500-1600 cm^-1^ and represents the amount of resonance in the carbon nanotubes. With the carboxylation of carbon nanotubes, the amount of resonance in f-MWCNT decreases and the G peak intensity decreases as well. Therefore in f- MWCNT, the ratio of the intensity of the D peak to the G peak (I_D_/I_G_) is higher than the naked MWCNT. In Figure 2, graphs a and b show the Raman spectra of MWCNT and f-MWCNT respectively. I_D_/I_G_ ratio of MWCNT is 1.86 and I_D_/I_G_ ratio of f-MWCNT is 2.26 which illustrates that treating with acid decreased the resonance and increased the amount of SP^3^ carbon in f-MWCNT. In Figure 2, graphs c and d show the Raman spectrum of MWCNT-MTX and MWCNT-MTX -PEI-FA respectively. By attaching compounds on the carbon nanotube surface, we expect that the vibrational frequency of the SP^2^ carbons will increase, therefore the G peak frequency must shift to higher frequencies. The vibration frequency of f-MWCNT is 1603 ± 0.57cm^-1^, while the frequency of vibration in MWCNT-MTX and MWCNT-MTX -PEI-FA is equal to 1607 ± 0.69 and 1607.2 ± 0.57 cm^-1^ respectively (Table 1), which proves that MTX, FA and PEI are fixed to the surface of carbon nanotubes. 

**Figure 3:**
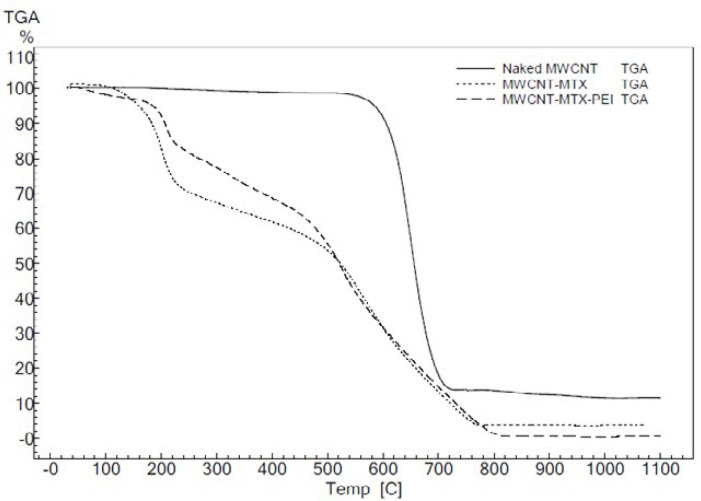
TGA of f-MWCNT, MWCNT-MTX and MWCNT-MTX- PEI-FA

Figure 3 shows the TGA of f-MWCNT, MWCNT-MTX and MWCNT-MTX-PEI-FA. The peaks corresponding to 200-400 °C in the MWCNT-MTX-PEI-FA and MWCNT-MTX spectra are associated with the destruction of FA, MTX and PEI on the surface of carbon nanotubes, as organic compounds are destroyed in this approximate temperature range.

Elemental analysis was used to determine the percentage of C, N and H elements. Table 2 shows the elemental analysis of different carriers. FA, MTX and PEI have nitrogen in their structure, therefore, we expect that by placing these compounds on f-MWCNT, the nitrogen content of these carriers will increase. The data in Table 2 shows that the nitrogen levels of MWCNT-MTX and MWCNT-MTX-PEI-FA are noticeably higher than that of f-MWCNT and MWCNT, which confirms the presence of nitrogen compounds on the MWCNT-MTX and MWCNT-MTX-PEI-FA surface.

For further confirmation of the presence of PEI, MTX and FA on the f-MWCNT surface, zeta potential analysis was used. Since PEI, MTX and FA possess amino groups, by placing these compounds on f-MWCNT, we expect the zeta potential of the carriers to shift positively. Table 3 shows the size, zeta potential, and polydispersity of f-MWCNT, MWCNT-MTX and MWCNT-MTX-PEI-FA. The zeta potential of MWCNT-MTX and MWCNT-MTX-PEI-FA is more positive than that of f-MWCNT, which shows that the compounds containing amino groups, namely PEI, MTX and FA, are positioned on the surface of f-MWCNT. PEI has a lot of amine groups compared with FA and MTX, therefore the zeta potential has become quite positive for MWCNT-MTX-PEI-FA. According to Table 3 the increase in the size of particles in MWCNT-MTX and MWCNT-MTX-PEI-FA compared with f-MWCNT are due to the presence of PEI, MTX and FA on the carbon nanotube surface. According to Table 3, the polydispersity index (PDI) of the carriers is good. PDI shows the particle size distribution and when PDI=0, the particles are monodispersed. 

**Figure 4:**
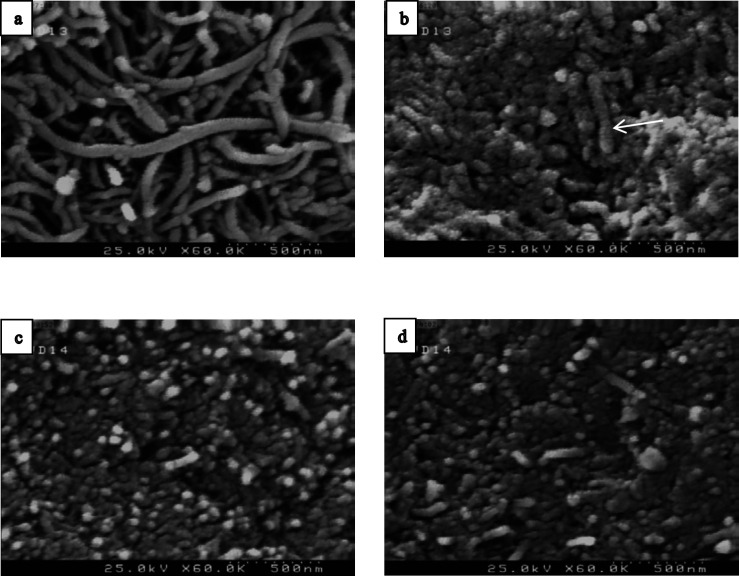
FE-SEM analysis of naked MWCNT (a), f-MWCNT (b), MWCNT-MTX (c) and MWCNT-MTX-PEI-FA (d).

Figure 4 shows the FE-SEM analysis of naked MWCNT, f-MWCNT and MWCNT-MTX and MWCNT-MTX-PEI-FA. Naked MWCNT (Figure 4a) has a length of several micrometers. Acidic treatment of the naked MWCNT reduces the size of carbon nanotubes. As is clear from Figure 4b, the particle size is below 500 nm. In Figure 4c and 4d, the surface of f-MWCNT has been modified, therefore, the size of the nanoparticles is not reduced.

*Release studies of MTX in MWCNT-MTX and MWCNT-MTX-PEI-FA*

**Figure 5:**
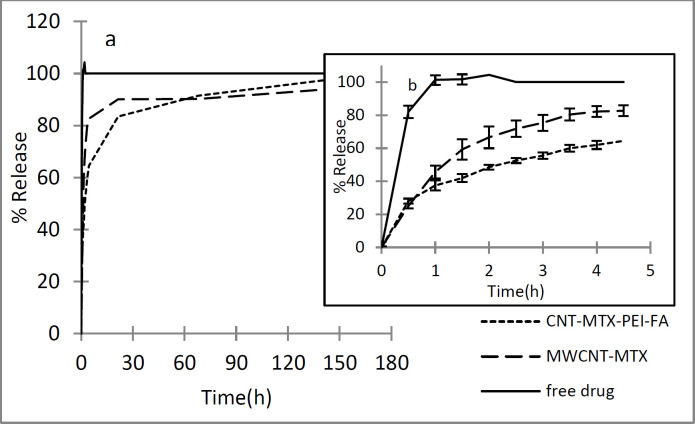
Release profile of MTX in free drug, MWCNT-MTX and MWCNT-MTX-PEI-FA in 180 h (a), and the same expriment in 5 h (b).

In order to investigate the release of methotrexate from MWCNT-MTX and MWCNT-MTX-PEI-FA, first, the wavelength in which MTX has maximum absorbance was obtained by the spectrophotometer. For this purpose, MTX absorbance spectrum was plotted at a concentration of 16 μg/mL in PBS (0.1 M and pH=7.4). The maximum absorbance (λ_max_) of MTX is at 300 nm. Therefore, MTX release is monitored at 300 nm. Figure 5 shows the release profile of free MTX at different times. As shown in Figure 5, the free drug is rapidly released, which indicates that the dialysis membrane is not a barrier against the release of free MTX and the detachment of MTX from MWCNT-MTX and MWCNT-MTX-PEI-FA determines the release rate. MTX release from MWCNT-MTX and MWCNT-MTX-PEI-FA is presented in Figure 5. For MWCNT-MTX, in the first 4.5 h, 83% of MTX is released due to high concentrations of MTX on the carrier surface which may cause toxic effects. In MWCNT-MTX-PEI-FA, the released MTX in the first 4.5 h is 65%. Thus, the release rate of MTX in MWCNT-MTX-PEI-FA is slower than that of MWCNT-MTX. This is due to the fact that branched polyethylenimine covers the surface of MWCNT-MTX-PEI-FA, therefore, methotrexate which is already detached from the carrier must first pass through the branches of PEI before being released. It can be concluded that placing the PEI-FA complex on the surface of MWCNT-MTX acts as a barrier against MTX release and reduces release rate.

In order to study the kinetics of release, zero order, first order, Higuchi, and Hixson Crowell kinetic models were utilized and the Korsmeyer- Pappas model was employed so as to analyze the mechanism of release and type of release of methotrexate. In zero order kinetics, the cumulative percentage of released drug is plotted against time, in first order kinetics, the natural logarithm of the concentration of unreleased drug is plotted against time, in the Higuchi model, the concentration of unreleased drug is plotted against the square root of time, and in the Hixson Crowell model, the third root of the concentration of unreleased drug is plotted against time. In order to investigate the mechanism of release, the Korsmeyer- Pappas method was employed and a graph displaying the logarithm of the concentration of released drug versus the logarithm of time was constructed. Table 4 shows the values of r^2^ for different models of methotrexate release from MXCNT-MTX and MWCNT-MTX-PEI-FA. It is clear from the data provided in Figure 4 that the release kinetics involved with MWCNT-MTX and MXCNT-MTX-PEI-FA are consistent with the Higuchi model. When determining the mechanism of release using the Korsmeyer- Pappas method, if the slope of the graph (n) is less than 0.45, the release mechanism is of fickian and dependent upon the concentration gradient of the drug. If the slope of the graph is between 0.45 and 0.89, the release mechanism is non- Fickian. The value of n for MWCNT-MTX and MWCNT-MTX-PEI-FA is calculated as 0.164 and 0.205 respectively which shows that the mechanism of release is fickian based.

*Cytotoxicity studies in the presence and absence of laser*

Carbon nanotubes are able to absorb a wide range of light from ultraviolet to infrared (IR) and convert it to heat, while biological systems are unable to absorb waves in the 700 to 1100 nanometer range. Therefore it is possible to send carbon nanotubes to the desired site in the body and subject that area to IR radiation. The carbon nanotubes absorb the laser’s lights and convert them into heat which leads to a rise in temperature in the target site. This feature may be used to eliminate cancer cells. For this purpose, folic acid is attached onto the surface of the carbon nanotube which causes the carbon nanotube to automatically target cancer cells and increases its uptake in cancerous cells compared with non-cancerous cells. The reason for this being a higher number of folic acid receptors on cancerous cells. With the increased uptake of folic acid, the carbon nanotubes also enter the cancer cells and accumulate inside of them. It is at this point that the cells undergo radiation from the lasers. The carbon nanotubes in the cells absorb this radiation and generate heat which in turn increases the temperature resulting in cell death. To augment this effect, another destructive agent such as methotrexate may be added onto the folic acid-carbon nanotube complex. This increases the anti-cancer effects of the carrier. In this study, an 808 nanometer continuous beam laser with a capacity of 1.5 W/cm^2^ was used. To carry out cellular toxicity studies, synthesized carriers in three final concentrations of 5, 10, and 20 mg/mL were added to the culture media of MCF7 cells and the toxicity of the carriers were investigated in the presence and absence of the laser. All cellular studies included a control group to which no carriers were added.

**Figure 6:**
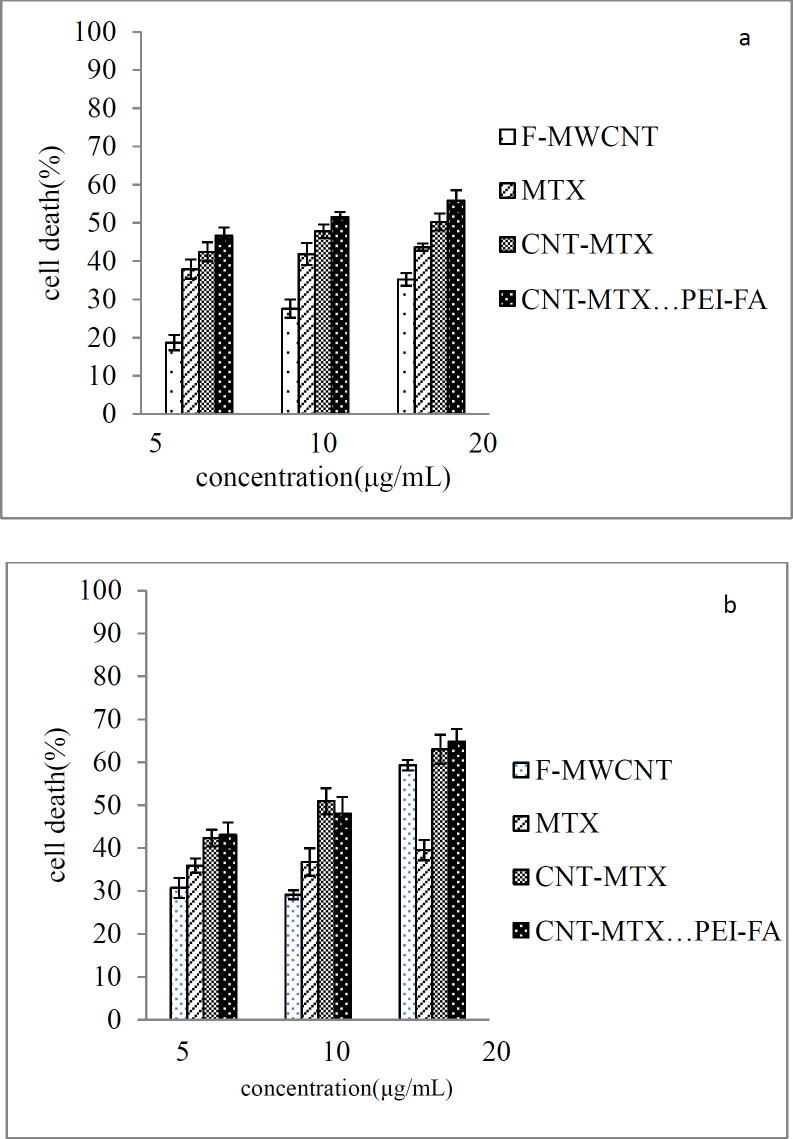
Cytotoxicity effect of MTX, f-MWCNT, MWCNT-MTX and MWCNT-MTX-PEI-FA in the absence (Figure 6a) and presence (Figure 6b) of laser

**Figure 7:**
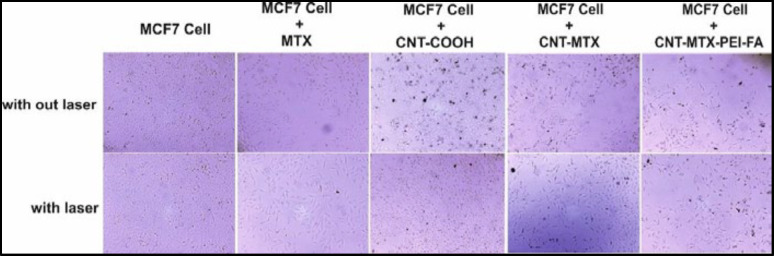
Cytotoxicity effect of MTX, f-MWCNT, MWCNT-MTX and MWCNT-MTX-PEI-FA in the absence and presence of laser on the MCF7 Cell

Figure 6a shows the cellular toxicity of MTX, f-MWCNT, MWCNT-MTX, and MWCNT-MTX-PEI-FA on MCF7 cells and Figure 7 shows the microscopic picture of the MCF7 cells. According to Figure 6a, in the absence of the laser, the highest number of cell death belongs to MWCNT-MTX-PEI-FA. The death of cells in contact with this carrier at a concentration of 20 mg/mL is equal to 56%. The reason for this being the cytotoxic effect of methotrexate coupled with the targeting effect of the carrier due to folic acid. The presence of folic acid on the carrier increases carrier accumulation on the cell surface, therefore, the carrier can be absorbed by cancer cells leading to increased cell death. The cell death associated with MWCNT-MTX at a carrier concentration of 20 mg/mL is equal to 50%. The decreased toxicity compared with MWCNT-MTX-PEI-FA is a result of MWCNT-MTX not being functionalized with FA and therefore being non-target specific. The cell death with f-MWCNT with the same concentration is equal to 35%. This is the least amount of toxicity in comparison with MWCNT-MTX-PEI-FA and MWCNT-MTX and can be attributed to a lack of methotrexate and folic acid. In other concentrations of the carrier, namely concentrations of 5 and 10 mg/mL, the observed trend is similar to the trend observed at 20mg/mL concentrations. The concentration of carrier that kills 50% of cells is referred to as IC50. The smaller the IC50 number, the more toxic the carrier is. Table 5 shows the IC50 values for MTX, f-MWCNT, MWCNT-MTX, MWCNT-MTX-PEI-FA. The smallest value for IC50 belongs to MWCNT-MTX-PEI-FA, because folic acid bestows specificity for target cells and methotrexate kills them, while MWCNT-MTX lacks a targeting agent and therefore has a higher IC50.

In order to determine whether or not there is a meaningful correlation between cell death variations with different carriers in the absence of a laser, a one way ANOVA test was performed. This test was performed on all four carriers (MTX, f-MWCNT, MWCNT-MTX, and MWCNT-MTX-PEI-FA) at three concentrations of 5, 10, and 20 mg/mL. A confidence interval of 95% was established as a basis for calculations. If the p-value is less than 0.05, the difference in cell death is considered meaningful. P-values for different carriers were calculated for all concentrations. The comparison between concentrations and variance analysis was done using LCD standard testing and results showed that there is a meaningful difference between the cellular toxicity attributable to the different carriers.

Figure 6b shows the cellular toxicity of MTX, f-MWCNT, MWCNT-MTX, and MWCNT-MTX-PEI-FA on MCF7 cells in the presence of a laser. It is clear from the graph that with the increase in concentration of each carrier, cell death also increases. In the presence of a laser and a concentration of 20 mg/mL, the highest amount of cell death belongs to f-MWCNT, MWCNT-MTX, and MWCNT-MTX-PEI-FA which is even greater than when there is no laser involved. For example, at a concentration of 20 mg/mL the percentage of cell death for the MWCNT-MTX carrier is 50% in the absence, and 63% in the presence of a laser. This is because, in the presence of a laser and high concentrations of the carbon nanotube, the carbon carriers have a strong photothermal effect. This means that the carbon nanotube absorbs the radiation from the laser and converts it to heat which increases temperature leading to cancer cell death. In the presence of a laser, in the case of the MWCNT-MTX and MWCNT-MTX-PEI-FA carriers, methotrexate also plays a role in cell death in addition to the photothermal effect. The combination of these two cytotoxic agents in the MWCNT-MTX and MWCNT-MTX-PEI-FA carriers makes it so that the cellular toxicity of the two carriers do not have a meaningful difference regardless of concentration. Statistical calculations using one way ANOVA test supported this hypothesis and showed that at any concentration in the presence of a laser, the amount of cell death for the MWCNT-MTX and MWCNT-MTX-PEI-FA carriers do not bear a meaningful distinction (p-value > 0.05). This is because the photothermal effect in combination with the cytotoxic effect of methotrexate results in a strong killing effect which renders concentration variations in the carriers virtually insignificant. This is why the cytotoxic effect for both MWCNT-MTX and MWCNT-MTX-PEI-FA is equal regardless of concentration.

On the other hand, the two MWCNT-MTX and MWCNT-PEI-FA carriers show a significant difference in amount of cell deaths compared with the two MTX and f-MWCNT carriers in the presence of a laser. The reason for the MTX carrier causing a lower percentage of cell death compared with the other carriers is that MTX is not affected by the laser and does not have photothermal effect. The reason that f-MWCNT causes a lower percentage of cell death compared with the MWCNT-MTX and MWCNT-MTX-PEI-FA carriers is that f-MWCNT lacks methotrexate. Though it should be noted that f-MWCNT exhibits a lower cell death percentage at concentrations of 5 and 10 compared to MTX despite having photothermal effect in the presence of a laser. This can be explained by the fact that at high concentrations of carbon nanotubes, the photothermal effect is amplified because the photothermal effect is extremely dependent upon concentration.

**Figure 8:**
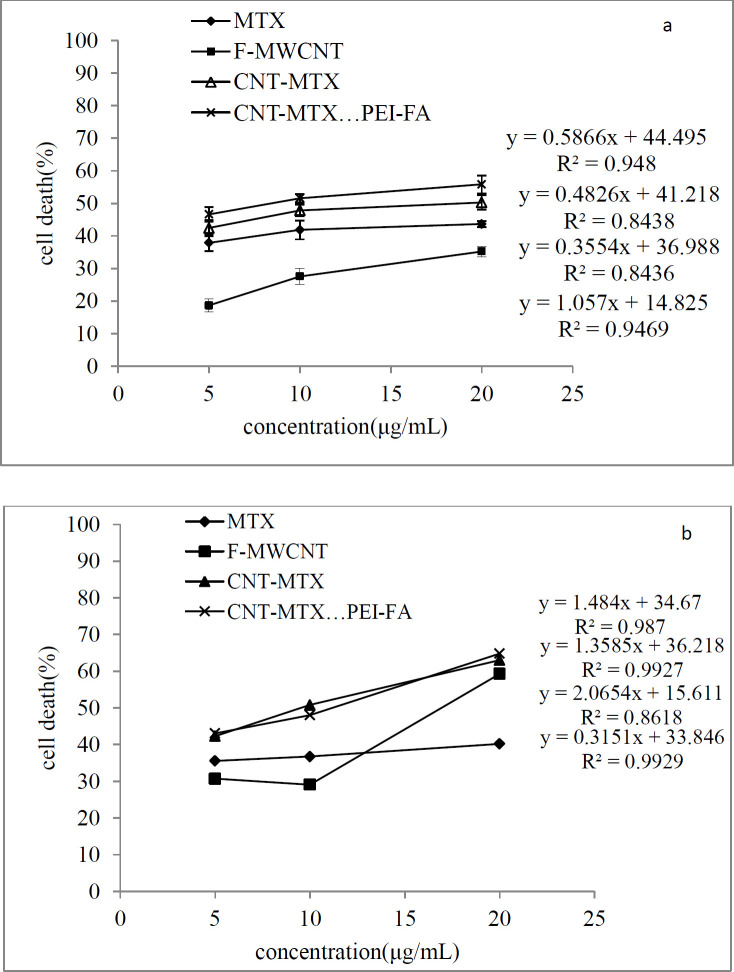
The cytotoxicity effect of MTX, f-MWCNT, MWCNT-MTX and MWCNT-MTX-PEI-FA in various concentrations (a) in the absence and (b) presence of a laser

The amount of cell death is expected to increase with an increase in carrier concentration. Figure 8 is a linear graph showing the effect of different concentrations of the MTX, f-MWCNT, MWCT-MTX, MWCNT-MTX-PEI-FA carriers on the death of MCF7 cells in the presence of (a), and the absence of (b), a laser. It can be deduced from Figure 8 that an increase in carrier concentration leads to an increase in the amount of cell death. Even though the presence of a laser does not affect cell death caused by MTX, it still increased the amount of cell death caused by carbon carriers especially at high concentrations. Regression analysis was used in order to investigate if there is a significant correlation between the increased concentration of carriers and cell death. The results showed that for all carriers, the value of p for the slope is less than 0.05 (*p*-value < 0.05), which signifies that there is a meaningful relationship between increasing carrier concentrations and cell death.

*Targeting* *studies in the presence of a laser*

There is a larger number of receptors for folic acid on cancer cells compared with healthy cells. This is why cancer cells are more likely to absorb folic acid compared with healthy cells. Therefore it is possible to use folic acid to deliver ligands and various drugs to cancer cells. The synthesized carrier MWCNT-MTX-PEI-FA possesses folic acid on its surface so it is expected that it will have greater uptake by cancer cells compared with the MWCNT-COOH and MWCNT-MTX carriers. With the accumulation of carriers inside the cancer cells, the cells are able to absorb infrared radiation from the laser, while without the carriers, they cannot absorb the laser emissions. The carbon nanotubes absorb the laser light and turn them into heat, which is a feature that can be used to kill cancer cells.

To investigate targeting potential, the MWCNT-MTX-PEI-FA, f-MWCNT, and MWCNT-MTX carriers were added separately with a concentration of 20 mg/mL each to the culture media of MCF7 cells. The cells containing these carriers were incubated for 3 h and the cells were washed twice with normal saline in order to remove carriers that did not attach to the cells. After that, 200 mL of fresh culture media was added to each well and immediately treated for 3 min with an 808 nm laser and incubated at 37°C for 48 h to determine cell death amount using MTT assay. If carriers are connected on the cancer cells, the temperature rises when coming into contact with the laser emissions and this leads to cell death. Because the MWCNT-MTX-PEI-FA carrier is more target specific compared with the rest of the carriers, we expect a higher number of cell death. The amount of cell death with f-MWCNT is equal to 37 ± 0.70 percent which shows that f-MWCNT can be absorbed by cancerous cells. This number is equal to 49.64 ± 2.44 percent for MWCNT-MTX which is due to the presence of MTX. The amount for MWCNT-MTX-PEI-FA is 55.11 ± 1.97 percent due to the target specific features of the carrier as well as the presence of MTX in the carrier. The results of this analysis is presented in Table 6. It can be concluded from the results that the cell death associated with the MWCNT-MTX-PEI-FA carrier is higher compared with the other two carriers and this carrier is more target specific than the others. Statistical studies on the cell death values have shown that all cell death values are significant and *P *< 0.05.

**Table 1.** Frequency of G peak

Carrier	G peak frequency
f-MWCNT	1603 ± 0.57
MWCNT-MTX	1607 ± 0.69
MWCNT-MTX-PEI-FA	1607.2 ± 0.75

**Table 2:** Elemental analysis data

% H	% N	% C	Carrier
**0.41**	0.59	92	MWCNT
**0.81**	0.09	80.20	f-MWCNT
**4.23**	12.10	70.54	MWCNT-MTX
**4.68**	11.66	70.09	MWCNT-MTX-PEI-FA

**Table 3:** Size, zeta potential, and polydispersity of carriers

quality	PDI	Zeta p	Z average(nm)	Carrier
**Good**	0.225	33.8-	117.1	f-MWCNT
**Good**	0.203	27.6-	156.2	MWCNT-MTX
**Good**	0.241	21.9+	281	MWCNT-MTX-PEI-FA

**Table 4:** Different models of MTX release in MWCNT-MTX and MWCNT-MTX-PEI-FA

** carrier**	**MWCNT-MTX**	**MWCNT-MTX-PEI-FA**
**kinetics**		
	r^2^	r^2^
Zero order	0/202	0/47
first order	0/497	0/903
Higuchi matrix	0/985	0/995
Hixson Crowell	0/377	0/771
Korsmeyer-Pappas	0/546	0/889

**Table 5:** IC_50_ of carriers in the absence of laser (n = 3).

**Carrier**	**F-MWCNT**	**MTX **	**CNT-MTX**	**CNT-MTX-PEI-FA**
IC_50_ (μg/mL)	31.50 ± 0.91	32.91 ± 0.91	16.98 ± 1.07	9.89 ± 0.38

**Table 6:** Percentage of cell death associated with f-MWCNT, MWCNT-MTX and MWCNT-MTX-PEI-FA

Carrier	Cell death
f-MWCNT MWCNT-MTX	37±0.70 49.64±2.44
MWCNT-MTX-PEI-FA	55.11±1.97

## Conclusions

In this study, MWCNT-MTX and MWCNT-MTX-PEI-FA were synthesized. The release profile of MTX in these two carriers showed that the presence of PEI on the surface of MWCNT-MTX acts as a barrier against release and reduces the MTX release rate. Cytotoxic studies showed that by increasing the concentration of the carriers, their cytotoxicity also increased. In the absence of a laser, MWCNT-MTX-PEI-FA exhibits the highest degree of cytotoxicity due to folic acid acting as a targeting agent leading to effective uptake by the MCF7 cells. In the presence of a laser, the cytotoxicity of MWCNT-MTX and MWCNT-MTX-PEI-FA has no significant difference, because the synergistic effect of methotrexate and photothermia in cell death is stronger than the other factors. Targeting studies have shown that MWCNT-MTX-PEI-FA can be absorbed by cancer cells exclusively.
